# Global Food Demand Scenarios for the 21*^st^* Century

**DOI:** 10.1371/journal.pone.0139201

**Published:** 2015-11-04

**Authors:** Benjamin Leon Bodirsky, Susanne Rolinski, Anne Biewald, Isabelle Weindl, Alexander Popp, Hermann Lotze-Campen

**Affiliations:** 1 Dept. of Climate Impacts and Vulnerabilites, Potsdam Institute for Climate Impact Research, Potsdam, Germany; 2 Commonwealth Scientific and Industrial Research Organisation, St. Lucia, Australia; 3 Dept. of Sustainable Solutions, Potsdam Institute for Climate Impact Research, Potsdam, Germany; 4 Dept. of Agricultural Economics, Humboldt University of Berlin, Berlin, Germany; Swedish University of Agricultural Sciences, SWEDEN

## Abstract

Long-term food demand scenarios are an important tool for studying global food security and for analysing the environmental impacts of agriculture. We provide a simple and transparent method to create scenarios for future plant-based and animal-based calorie demand, using time-dependent regression models between calorie demand and income. The scenarios can be customized to a specific storyline by using different input data for gross domestic product (GDP) and population projections and by assuming different functional forms of the regressions. Our results confirm that total calorie demand increases with income, but we also found a non-income related positive time-trend. The share of animal-based calories is estimated to rise strongly with income for low-income groups. For high income groups, two ambiguous relations between income and the share of animal-based products are consistent with historical data: First, a positive relation with a strong negative time-trend and second a negative relation with a slight negative time-trend. The fits of our regressions are highly significant and our results compare well to other food demand estimates. The method is exemplarily used to construct four food demand scenarios until the year 2100 based on the storylines of the IPCC Special Report on Emissions Scenarios (SRES). We find in all scenarios a strong increase of global food demand until 2050 with an increasing share of animal-based products, especially in developing countries.

## Introduction

Over the last 50 years, global food demand approximately trippled [1]. This rapid growth was caused on the one hand by the doubling of world population from about 3 to more than 6 billion people and on the other hand increased per-capita demand due to rising living standards. In this article, we define food demand as equivalent to food supply according to FAOSTAT [1]. It describes the daily average availability of food in households in kcal capita^−1^ d^−1^, comprising both food intake and non-eaten household food waste. Detecting a basic rule of microeconomics, the German statistician Engel observed already in the middle of the 19th century a positive correlation between per capita food demand and income that levels out for high incomes, the so called “Engel’s Law” [2]. Yet beside income, per capita demand is also influenced by other factors such as climate, age and gender composition of the population, food prices, development of the food industry, globalisation and openness to global markets, the degree of urbanisation, or changes in the role of women in society [3–5].

At the same time, more and more animal-based products like meat, milk, eggs and fish are being consumed. In the last five decades, the global population-weighted average share of animal-based products rose from 15.4 to 17.7% (years 1961–2009, [1]). A closer look however reveals, that this increase can mainly be attributed to rising consumption of animal-based products in developing and emerging economies, while in developed countries this share stagnated or even decreased in the last decades [1]. As with total food demand, income is a major driver for the share of animal-based products, but also the development of the livestock industry, state interventions, food prices, cultural preferences, globalisation and openness to global markets, and health concerns are determining factors [3–5].

Food demand can be used as a simple indicator for food security, even though such an index has to be interpreted with caution. According to FAO’s definition [6], food security can be analyzed with respect to (1) food availability, (2) food stability over time, (3) access to food, including the potential of individuals to purchase food, and (4) utilization of food to create an adequate and healthy diet. Food demand as analyzed in this study is mostly an indicator for food availability, while one has to bear in mind that the FAO estimates do not cover food provision from hunting and gathering of wild products, which may supply a significant share of food calories to households in developing countries (see S1 Text). The information on food stability over time and access to food is partly lost as FAO estimates are averaged over one year and over the population of one country. For example, in the United States of America about 11% of households are not food secure for the whole year, and 3.5% suffer from hunger [7], even though the per-capita demand in the United States is with more than 3800 kcal capita^−1^ d^−1^ one of the highest in the world. Hence, food wastage and hunger can coexist due to distributional inequality.

Scenarios of future food demand are an important tool to develop strategies for achieving the goal of global food security. Scenarios are used when uncertainty is too high to allow for predictions or forecasts. Instead, they are alternative images of the future that “enhance our understanding of how systems behave, evolve and interact” [8]. They can be used to explore possible futures and to localize the regions where most effort is needed to combat hunger. Furthermore, they help to distinguish periods in which the problems can be expected to persist, such that strategies can be adapted accordingly.

Moreover, food demand scenarios are also used in different contexts, for example to estimate future cropland expansion [9], agricultural greenhouse gas emissions [10, 11], trade, technology and water prices [12], agricultural intensification [13], and the use of pesticides [14]. For many of these studies, the amount of animal-based calories may play a special role, as their production requires a multitude of plant calories and puts a high pressure on the land-use system and the environment [15, 16].

The rising computing power allows to explore more and increasingly diverse pathways into the future. Hereby, it becomes important that scenarios are easily adaptable to changing storylines to account for many possible future worlds. Additionally, flexibility regarding the aggregation of world regions and the possibility to expand the time horizon are desired.

A number of studies already estimate future food demand [17–22]. However, there are several shortcomings of previous studies. Firstly, there has been no study yet that derives future estimates without building on expert estimates. Some studies like Alexandratos & Bruinsma [17], Alexandratos et al. [18], and Dorin et al. [20] directly draw from data supported expert estimates. Other studies [19, 22] create quantitative estimates based on computable general equilibrium or partial equilibirum models. However, these demand models are designed primarily to simulate shocks on markets. For long-term scenarios, the parameters in the models have to be adjusted based on assumptions in order to derive caloric estimates for the future that are not unrealistically high or low [22]. Often, the parameters are adjusted in a way that the model results become consistent with the numbers in the FAO report “World agriculture towards 2030/2050” by Alexandratos & Bruinsma [17][22]. Consequently, there are no econometric model estimates yet available with the potential to challenge or support the expert estimates. Secondly, the available literature is not very transparent. It remains unclear how the expert-estimates were derived [17, 18], or which exact parameters, functional relations and calibration steps were used to create the model-based projections. Thirdly, most studies provide only data for a single storyline and cover a time-horizon not beyond 2050. However, users of food demand projections have a strong need for multiple scenarios to explore the sensitivity of their analyses to changes in dietary patterns. Furthermore, climate impact research requires scenarios until 2100 [8], which will inevitably show a much wider range of uncertainties between different storylines. Fourthly, the results are not reproducible, and scenarios cannot be easily adapted to the needs of scientific communities that use the projections as input data.

Therefore, we provide a transparent method for creating specific food demand scenarios for total and animal-based calories, requiring only population and income projections as input, while no information on the food supply side is needed. This tool shall support the work of other scientists who require food demand scenarios as input for policy-relevant studies on food security, environmental assessments, or agricultural outlooks. The scenarios that can be created are flexible regarding the time horizon and provide information on the national level, which can be aggregated to regions of any size and location. Our method is exemplarily applied to the storylines of the SRES scenarios [8] to create country-based projections until the year 2100. The SRES storylines are broadly used in the literature and were further elaborated in the Millenium Ecosystem Assessments [23, 24]. Beyond, our method could be applied to many other scenario projects: To the climate-related global IPCC storylines SA90 [25], IS92 [26] or the upcoming SSP [27] and SPA storylines [28]; to general long-term scenarios for sustainability research like the Club of Rome scenarios [29] or the Millenium Ecosystem Assessments [23, 24]; or to scenarios on agriculture and land-use that are focussed on specific world regions like [30]. They could also help as a tool for participatory scenario-building processes like CCAFS [31].

## Materials and Methods

Our approach uses country-specific historical data to fit regressions of food demand on income over time which we apply to scenarios of future income and population.

### Data sources

We use data on historical food demand provided by the United Nations Organisation for Food and Agriculture (FAO) in the FAOSTAT database (Table 1). Demand is given in kcal capita^−1^ d^−1^ per country from 1961 to 2007 as total calorie (*C*_*T*_) and animal-based calorie demand (*C*_*L*_). The data include a wide range of unprocessed and processed agricultural and fishery products intended for food consumption.

**Table 1:** Data sources. ItC: Item code, EC: Element code, IC: Indicator code. Download of FAO data from http://faostat.fao.org on 27.10.2010 and from Worldbank from http://data.worldbank.org on 13.09.2011.

Value	Unit	Years	Codes	Sources
*C*_*T*_	kcal capita^−1^ d^−1^	1961–2007	ItC: 2901, EC: 664	FAOSTAT
*C*_*L*_	kcal capita^−1^ d^−1^	1961–2007	ItC: 2941, EC: 664	FAOSTAT
*I*	US$_2005_ capita^−1^ a^−1^	1960–2007	IC: NY.GDP.PCAP.KD	WDI
*P*	capita	1960–2007	IC: SP.POP.TOTL	WDI
$I_{t,c}^{Proj}$	US$_1990_ capita^−1^ a^−1^	1990–2100	A1, A2, B1, B2	CIESIN
$P_{t,c}^{Proj}$	capita	1990–2100	A1, A2, B1, B2	CIESIN

Historical data on population and per-capita income are taken from the World Bank in the World Development Indicator (WDI) database (Table 1, [32]). Population (*P*_*c*_) is given in capita per country *c* and income values (*I*_*c*_) [gdp capita^−1^ year^−1]^ in constant US$_2000_ from 1961 to 2007, based on market exchange rate (MER). For income (*I*) and calorie demand (*C*_*T*_,*C*_*L*_) there are 5889 complete data pairs from 162 countries.

Projections of per capita income ($I_{t,c}^{Proj}$) and population ($P_{t,c}^{Proj}$) are based on the SRES storylines [8]. Population scenarios were estimated with the UN (A2, B2) and the IIASA model (A1, B1). Four storylines (A1, A2, B1, B2) are distinguished based on diverging assumptions on the societal development concerning economical values as well as regional connectivity. We used the country-specific datasets from CIESIN database [33, 34]. population projections ($P_{t,c}^{Proj}$) are given in capita (Figure A in S2 Fig) and per-capita income projections ($I_{t,c}^{Proj}$) in US$_1990_ capita^−1^ a^−1^ (Figure B in S2 Fig) for the years 1990–2100 in 5-year increments. For consistency of historical and projected income values, both were transformed into US$_2005_ using deflators from the World Bank [32].

### Functional relations between income and food demand

Different functional relations can be formulated between food demand, income *I* and time *t*. Here, we estimate the parameters of two functional relations for both total per capita demand and for the share of animal-based calories. These relations can subsequently be used to customize scenarios to their storylines.

Data preparation and analysis is performed using the programming language and statistical software R [35]. To select the necessary predictors, regressions are compared by an analysis of variance and stepwise reduction of the models is performed using the Akaike Information Criterion AIC (function *step* of package stats). The resulting models were tested against observed data with linear regressions using function *lm* of package stats.

#### Total calorie demand

For total calorie demand *C*_*T*_, we apply the Engel curve with an under-proportional increase with income *I*. The relation can be described by the power function *g* (Eq 1) with parameter *β* which can be interpreted as an elasticity (see review in [36]).

$$g\left(I\right)=C_{T}=\alpha \cdot I^{\beta }.$$ (1)

We model the parameters *α* and *β* to be time-dependent which represents the influence of other drivers than income. In order to provide appropriate functions for the scenario storylines, parameters *α* and *β* are determined in two alternative ways. For one parameter set we assume a linear temporal relation, and for the second set a decreasing time increment.

The first parameter set assumes a linear temporal relation and uses the linearized formulation of Eq (1)

$$\ln\left(g\left(I\right)\right)=\ln\left(C_{T}\right)=\ln\left(\alpha \right)+\beta \cdot \ln\left(I\right)\;\;.$$ (2)

Parameters α* = ln(*α*) and *β* are estimated by a linear mixed-effect model (using function *lmer* of package lme4, [37]). Hereby, ln(*C*_*T*_) is described with the fixed effect ln(*I*) and random effect time *t*. This results in time-dependent relations (exponential for *α* and linear for *β*) and is further on referred to as formulation *g*_*A*_ (Eq 3).

$$g_{A}\left(I,t\right)=exp\left(\alpha_{1}+\alpha_{2}\cdot t\right)\cdot I^{\left(\beta_{1}+\beta_{2}\cdot t\right)}\;\;.$$ (3)

The second parameter set assumes a decreasing time increment. Parameters *α* and *β* are estimated per year by a nonlinear least squares model fitted to Eq (1) (using function *nls* of package stats, [38]). Subsequently, the time series of parameters *α* and *β* are fitted to the asymptotic function *m* of Michaelis-Menten type (Eq 4) with intercept *ω*_1_, asymptotic value *ω*_2_, half-saturation constant *ω*_3_ and base year *t*_1_ = 1960.

$$m\left(t\right)=\omega_{1}+\frac{\omega_{2}\cdot \left(t-t_{1}\right)}{\left(t-t_{1}+\omega_{3}\right)}.$$ (4)

With the resulting functions *m*_*α*_ and *m*_*β*_, the alternative formulation for *g* can be written as

$$g_{B}\left(I,t\right)=m_{\alpha }\left(t\right)\cdot I^{m_{\beta }\left(t\right)}\;\;.$$ (5)

This captures the dynamics with rapidly changing values during the first 20 years of observations and rather less increase in the last period and is referred to as formulation *g*_*B*_ (Eq 5).

Both functional relations *g*_*A*_ and *g*_*B*_ can be applied to the historical data in a way that assumptions on their future development can be chosen according to the storyline of the respective scenario.

#### Animal calorie share

To estimate the animal-based calorie demand *C*_*L*_, the share of animal-based calories *C*_*LS*_ (Eq 6) in total calories *C*_*T*_ is taken.

$$C_{LS}=\frac{C_{L}}{C_{T}}$$ (6)

Again, two functional relations are used to estimate the demand according to the chosen storylines. First, the linear regression *h*_*A*_ (Eq 7) on income *I* and time *t* is fitted on logarithmic values of *C*_*LS*_ as

$$h_{A}\left(I,t\right)=C_{LS}=\exp\left(\kappa_{1}+\kappa_{2}\cdot \ln\left(I\right)+\kappa_{3}\cdot t+\kappa_{4}\cdot \ln\left(I\right)\cdot t\right)\;.$$ (7)

This approach assumes that livestock products are a normal necessity good in accordance with Bennet’s law [39].

Secondly, we assume that animal products are a normal necessity good at low incomes, but turn into inferior goods at high incomes. This can be justified e.g. by the ambition of better educated people for a more balanced, healthy or sustainable diet [36]. For the derivation of *h*_*B*_ (Eq 8), the under-proportionally increasing square root function is multiplied with an exponential decline. Thus, the share of animal-based calories declines at high incomes but does not become negative.

$$h_{B}\left(I,t\right)=C_{LS}=\left(\rho_{1}+\rho_{2}\cdot t\right)\cdot \sqrt{I}\cdot \exp\left(-\left(\lambda_{1}+\lambda_{2}\cdot t\right)\cdot I\right)$$ (8)

The functional relations *h*_*A*_ and *h*_*B*_ will be chosen for the projections of *C*_*LS*_ according to the storylines.

### SRES scenario preparation

Generally, scenario construction is focussed on variables and assumptions that are both subject to the largest uncertainty and that have major impacts on the results. For our analysis, we vary the assumptions on population growth, on income growth, and on the functional relations between per-capita income and dietary consumption patterns. The latter reflects the process uncertainty of dietary dynamics, and highlights the ambiguity in the past data that is consistent with various model representations.

#### Choice of functional relations based on storylines of SRES scenarios

Storylines have the purpose to harmonize scenario parameters, as in our case the assumptions on population growth, income growth and functional relations. The four storylines used in this study were developed to analyze the impacts of global climate change and possible mitigation strategies [8]. The A1 and A2 scenarios describe worlds emphasizing economic growth with societies that are characterized by materialistic attitudes. In contrast, the B1 and B2 scenarios describe worlds developing towards a sustainable economy, and where the society has a stronger environmental awareness. Common features of the A1 and B1 scenarios are a globalized economy and increased cultural and social interactions, whereas A2 and B2 storylines emphasize more self-reliance and local solutions to economic and social problems. A1 and B1 are therefore assumed to have a higher level of education and labor productivity, which also translates into lower reproduction rates and therefore slower population growth or even decreasing population. The higher population growth in A2 and B2 scenarios results in relatively lower per-capita incomes as the available resources have to be shared by a larger population. With regard to dietary habits, the SRES storylines suggest that the A1 and A2 scenarios describe a society with an affluent, wasteful and materialistic consumption pattern, even though dynamics like increased health consciousness in rich countries might also here reduce the consumption of animal-based calories. In contrast, the B1 and B2 worlds have societies with high environmental awareness that translate into a de-materialization of consumption, a reduced intake of animal-based calories and lower rates of household waste.

In accordance with the storylines, population and income projections were selected from the SRES database. Moreover, to represent the differences in the materialistic A1 and A2 storylines and the sustainable B1 and B2 storylines, we assign different functional relations for A1 and A2 than for B1 and B2. The globalized worlds A1 and B1 and their fragmented counterparts A2 or B2 are only distinguished by different population and income growth paths. As selection criterion for the allocation of the functional relation, we used the level of consumption that will prevail in the long-run in high income countries. The levels of consumption are higher in the *g*_*A*_ and *h*_*A*_ functions, that were applied to the materialistic A1 and A2 scenarios (Eq 9). For the storylines with a focus on environmentally sustainable lifestyles (B1 and B2), the formulations *g*_*B*_ and *h*_*B*_, that result in a much lower consumption in the long-run, were chosen (Eq 10). All functional relations are consistent with past data; the differences just reflect divergent interpretations of the data.

$$A_{1},{\text{A}}_{2}:C_{T}=g_{A}\left(I,t\right),C_{LS}=h_{A}\left(I,t\right)$$ (9)

$$B_{1},{\text{B}}_{2}:C_{T}=g_{B}\left(I,t\right),C_{LS}=h_{B}\left(I,t\right)$$ (10)

#### Calculating the demand scenarios

The data sources from FAOSTAT [1], Worldbank [32], and CIESIN [33, 34] do not completely cover all countries and the full time-span. For the scenarios (not for the regression analysis), we harmonized the data sources and filled missing data with regional average values. We excluded all countries not listed in FAOSTAT [1], representing 2.1% of the world population according to Worldbank [32]. Historical GDP data was completed with estimates of UNSTATS [40] for the countries of the Eastern Bloc.

For the 4 SRES scenarios, we projected per-capita vegetal ($C_{t,c}^{V\_Proj}$, Eq 11) and animal-based calorie demand ($C_{t,c}^{L\_Proj}$, Eq 12) for time *t* and countries *c* based on income $I_{t,c}^{Proj}$.

$$C_{t,c}^{V\_Proj}=g\left(I_{t,c}^{Proj}\right)\cdot \left(1-h\left(I_{t,c}^{Proj}\right)\right)\;\;\forall \;t,c$$ (11)

$$C_{t,c}^{L\_Proj}=g\left(I_{t,c}^{Proj}\right)\cdot h\left(I_{t,c}^{Proj}\right)\;\;\forall \;t,c$$ (12)

To meet the historical data for $C_{t,c}^{V\_Proj}$ and $C_{t,c}^{L\_Proj}$, we calibrated our estimates to the observed values $C_{1990,c}^{V\_cal}$ and $C_{1990,c}^{L\_cal}$ in the year 1990. There is evidence that heterogeneous national dietary patterns become more homogeneous through globalization (see e.g. [41–43]). Our regression being a plausible target for a globalized diet, we assume that the deviations of observed values from our regression function gradually disappear until 2100. For this purpose, we applied the function *conv* (Eq 13), for which calibrated demand *X*_*t*_ meets historical values *C*_0_ in an initial year *y*_*s*_ and converges to the regression values until the final year *y*_*e*_.

$$conv\left(X_{t},C_{0},y_{s},y_{e}\right)=\{\begin{array}{cc} \frac{t-y_{s}}{y_{e}-y_{s}}X_{t}+\frac{y_{e}-t}{y_{e}-y_{s}}C_{0} & \forall \;t\;\in \;[y_{s},y_{e}]\\ X_{t} & \forall \;t>y_{e} \end{array}$$ (13)

$$C_{t,c}^{V\_calib}=conv\left(C_{t,c}^{V\_Proj},C_{1990,c}^{V\_cal},1990,2100\right)\;\;\forall \;t,c$$ (14)

$$C_{t,c}^{A\_calib}=conv\left(C_{t,c}^{L\_Proj},C_{1990,c}^{L\_cal},1990,2100\right)\;\;\forall \;t,c$$ (15)

Finally, the calibrated per-capita estimates were multiplied with the population projections $P_{t,c}^{Proj}$ to obtain total vegetal demand $E_{t,c}^{V\_Proj}$ (Eq 16) and total animal-based demand $E_{t,c}^{L\_Proj}$ (Eq 17).

$$E_{t,c}^{V\_Proj}=C_{t,c}^{V\_calib}\cdot P_{t,c}^{Proj}\;\;\forall \;t,c$$ (16)

$$E_{t,c}^{L\_Proj}=C_{t,c}^{A\_calib}\cdot P_{t,c}^{Proj}\;\;\forall \;t,c$$ (17)

For regional aggregates, every country was assigned to one of ten world regions (S1 Fig) [44].

The validity of the projections was assessed by deriving trends for the transitional period from 1990 to 2007 using the partial Mann-Kendall test [45, 46]. The trend itself is determined as median value of all differences between the values and their successors. Trends were considered to be significant for *p* < 0.05.

### Sensitivity analysis of demand regression models

In order to provide information on the robustness of the estimated parameters, we performed a sensitivity analysis with respect to the underlying data. For each of the regression models, a random selection of 67% of the data pairs was chosen 1000 times and the respective regression parameters were estimated. The parameters were divided by the respective values derived from the entire dataset. For these relative factors, median and standard deviation were calculated. In order to evaluate the robustness of the regression models, the goodness of fit of dataset versus model estimates was calculated for the part of the dataset not used for the regression.

## Results

For both total calorie demand and animal-based calorie shares, results are presented for the two alternative formulations. Subsequently, features of food demand projections following the SRES storylines are described. We concentrate on regional results, while country-level estimates are given in the Supplement (S4, S5, S6 and S7 Figs). An interactive web-application can be used to explore the regional results (given in the Supporting Information). Own scenarios can be generated using the package “CalorieDemand” in the open source programming language R [35] (Supporting Information).

### Regressions for total calorie demand from historical data

**Fig 1:**
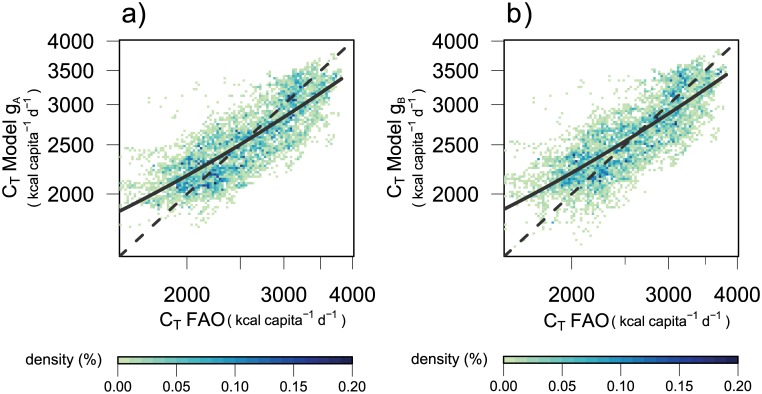
Model estimation for total calorie demand *C*_*T*_ a) with formulation *g*_*A*_ (Eq (3), SRES scenarios A1 & A2) and b) with formulation *g*_*B*_ (Eq (5), SRES scenarios B1 & B2). Comparison of reported and modelled total calories given as frequency in percent of the number of data pairs (colorscale below). Lines denote the linear regression (solid, values see Table 3) and 1:1 line (dashed). Note log scale of axes.

**Fig 2:**
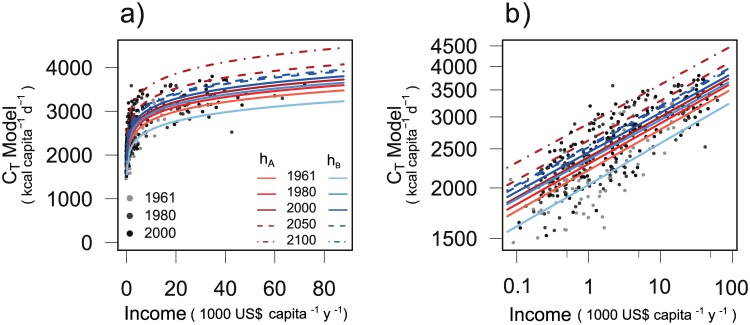
Model estimation for total calorie demand *C*_*T*_ with formulations *g*_*A*_ (red colors) and *g*_*B*_ (blue colors). Predictions for years 1961, 1980, 2000, 2050 and 2100 (lines) as well as reported data for 1961, 1980 and 2000 (dots) with a) linear axes scale and b) logarithmic axes scale.

For the formulation *g*_*A*_ for total calorie demand, the linear mixed model of log-transformed data (Eq 3) determines linearly increasing values for the time-dependent parameter α* and linearly decreasing values for parameter *β* (Figure A in S3 Fig). The coefficients of determination of the linear regressions for both parameters are higher than 0.95 (Table 2). Parameter *β*, the income elasticity of the demand, is estimated by the mixed model as 0.1002 ± 4.37 ⋅10^−4^ (mean ± SE) and decreases from 0.101 in 1961 to 0.0995 in 2007. The overall fit of the model to observed data (Fig 1a) is highly significant with a coefficient of determination of 0.65 and slope 0.66 (Table 3). The temporal development of the model (Fig 2) at high income *I* = 60,000 US$ shows an increase from 3587 kcal capita^−1^ d^−1^ in 2000 to 4289 kcal capita^−1^ d^−1^ in 2100.

**Table 2:** Regression parameters with linear time dependence for total calories for formulation *g*_*A*_ (Eq 3). Significance levels for *p*-values are denoted by (***): < 0.001, (**): ∈ [0.001, 0.01), (*): ∈ [0.01, 0.05), (.): ∈ [0.05, 0.1).

	Intercept	Slope	r^2^	*p*-value	F-statistics
α*	2.825	2.131⋅10^−3^	0.96	< 0.001 (***)	1057
*β*	0.162	-3.124⋅10^−5^	0.96	< 0.001 (***)	1057

**Table 3:** Statistical properties of regression models on total calorie demand (*C*_*T*_) and animal-based calorie share (*C*_*LS*_). Significance levels for *p*-values are denoted by (***): < 0.001, (**): ∈ [0.001, 0.01), (*): ∈ [0.01, 0.05), (.): ∈ [0.05, 0.1).

Model	Intercept	Slope	r^2^	*p*-value	F-statistics
*g*_*A*_ on *C*_*T*_	857.9880	0.658	0.65	< 0.001 (***)	11060
*g*_*B*_ on *C*_*T*_	846.4430	0.678	0.64	< 0.001 (***)	10551
*h*_*A*_ on *C*_*LS*_	0.0389	0.705	0.63	< 0.001 (***)	9913
*h*_*B*_ on *C*_*LS*_	0.0423	0.706	0.62	< 0.001 (***)	9685

For the formulation *g*_*B*_, we use regression models (Eq 5) with diminishing time-dependence of the parameters α* and *β* of Michaelis-Menten type (Eq 4). Parameters were determined significantly (all *p*-values < 0.001, Table 4) (Figure A in S3 Fig). Parameter *β* is estimated as 9.88 ⋅10^−2^ ± 3.21 ⋅10^−3^ (mean ± SE). The resulting demand values show a high correlation with observed data (Fig 1b, Table 3) with a comparable coefficient of determination as *g*_*A*_ and a slightly higher slope of 0.68. The temporal change is similar to *g*_*A*_ for the historical time span but nearly disappears for the projected years (Fig 2). At income values of *I* = 60,000 US$, total calorie demand after *g*_*B*_ changes from 3661 kcal capita^−1^ d^−1^ in 2000 to 3805 kcal capita^−1^ d^−1^ in 2100.

**Table 4:** Regression parameters with declining time dependence for total calories for formulation *g*_*B*_ (Eq 4) (RSE: residual standard error). Significance levels for *p*-values are denoted by (***): < 0.001, (**): ∈ [0.001, 0.01), (*): ∈ [0.01, 0.05), (.): ∈ [0.05, 0.1).

	*ω*_1_	*ω*_2_	*ω*_3_	*p*-value	RSE	Df
*α*	9.339⋅10^2^	3.875⋅10^2^	9.775	< 0.001 (***)	9.579⋅10^−7^	45
*β*	8.941⋅10^−2^	8.445⋅10^−3^	-7.557⋅10^−1^	< 0.001 (***)	4.654⋅10^−3^	45

### Regressions for the animal-based calorie share from historical data

The animal-based calorie demand is determined by using the proportion of animal-based to total calories *C*_*LS*_ and fitting the linear regression model *h*_*A*_ (Eq 7) and the inverted U-shaped curve *h*_*B*_ (Eq 8).

**Fig 3:**
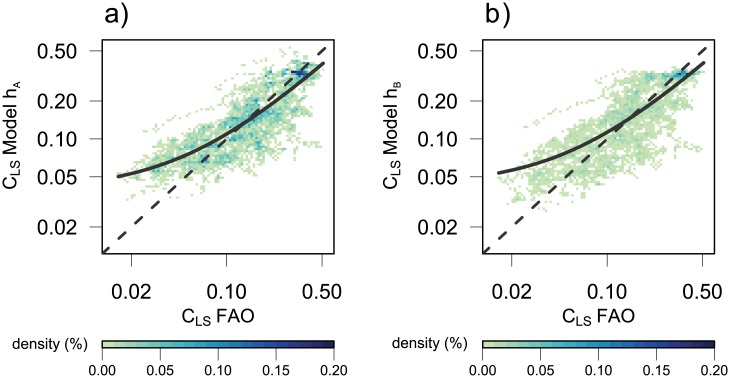
Same as Fig 1 for animal-based calorie share *C*_*LS*_ with formulation *h*_*A*_ (Eq (8), SRES scenarios A1 & A2) and *h*_*B*_ (Eq (8), SRES scenarios B1 & B2).

**Fig 4:**
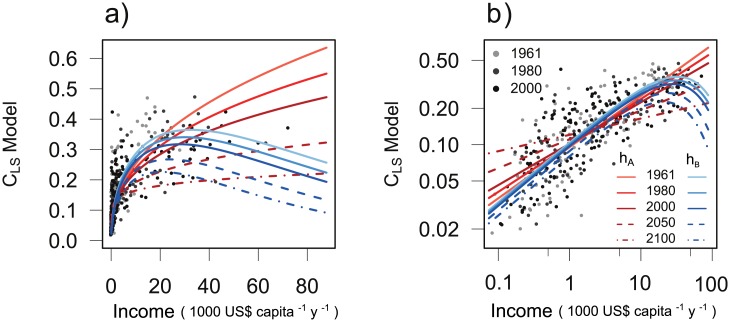
Same as Fig 2 for animal-based calorie share *C*_*LS*_ with formulation *h*_*A*_ (Eq (8), SRES scenarios A1 & A2) and *h*_*B*_ (Eq (8), SRES scenarios B1 & B2).

For the monotonically increasing function *h*_*A*_, anova results show that ln(*I*) and time *t* are significant predictors for ln(*C*_*LS*_) as well as the interaction of both. The linear regression model (Eq 7, parameters in Table 5) is highly significant (Table 3) with a slope of 0.63 and intercept 0.04 (Fig 3a). Predictions with this model (Fig 4) decrease dramatically at higher incomes of 60,000 US$ from 0.42 in 2000 to 0.2 in 2100. Although the coefficients of income and time are positive, the negative coefficient for the interaction produces this decline (Table 5).

**Table 5:** Regression parameters of the multiple linear regression for animal-based calorie share *h*_*A*_ (Eq 7). Significance levels for *p*-values are denoted by (***): < 0.001, (**): ∈ [0.001, 0.01), (*): ∈ [0.01, 0.05), (.): ∈ [0.05, 0.1).

Parameter	Value	*p*-value
*κ*_1_	-3.673⋅10^1^	< 0.001 (***)
*κ*_2_	4.497⋅10^0^	< 0.001 (***)
*κ*_3_	1.604⋅10^−2^	< 0.001 (***)
*κ*_4_	-2.077⋅10^−3^	< 0.001 (***)

The nonlinear function *h*_*B*_ (Eq 8) is fitted for time-dependent parameters *ρ* and *λ* (Table 6). With the estimated values, parameter *ρ* = *ρ*_1_ + *ρ*_2_ ⋅ *t* is decreasing and *λ* = *λ*_1_ + *λ*_2_ ⋅ *t* is increasing over time (Figure B in S3 Fig). Thus, for a given income level both parameters affect the values over time in a declining way. This model is highly significant (Fig 3b) with a slightly higher coefficient of determination of 0.62 (Table 3) and less overestimated values than with the multiple regression (compare Fig 3a). Predictions in lower income ranges up to 20,000 US$ are comparable to the multiple regression model *h*_*A*_ but give much lower values at higher incomes (Fig 4). At 60,000 US$, the animal-based calorie share drops from 0.28 in 2000 to 0.14 in 2100.

**Table 6:** Regression parameters for animal-based calorie share *h*_*B*_ (Eq 8). Significance levels for *p*-values are denoted by (***): < 0.001, (**): ∈ [0.001, 0.01), (*): ∈ [0.01, 0.05), (.): ∈ [0.05, 0.1).

Parameter	Value	*p*-value
*ρ*_1_	1.372⋅10^−2^	< 0.001 (***)
*ρ*_2_	-5.295⋅10^−6^	< 0.010 (**)
*λ*_1_	-1.102⋅10^−4^	0.062 (.)
*λ*_2_	6.404⋅10^−8^	0.031 (*)

### Projections for the total calorie demand

The projections of the SRES scenarios already start in the year 1990 such that the resulting demand values can be compared to historical data in an overlapping period of 20 years (Tables 7 and 8 in section on comparison of results). Total calorie demand in all country-based projections increases but distinct differences appear between scenarios A1 (S4 Fig) and A2 (S5 Fig) and those of B1 (S6 Fig) and B2 (S7 Fig), especially in Sub-Saharan Africa. Also for industrial countries in Europe or Northern America, the level as well as the proportion of demand values to those in transitional countries in Asia and southern America differ.

**Table 7:** Significant trends (values only shown for p < 0.05) for total calorie demand (kcal capita^−1^ d^−1^ a^−1^) between 1988 and 2007 for FAO data and between 1990 and 2010 for all scenarios estimated using the Mann-Kendall test.

Region	FAO	A1	A2	B1	B2
AFR	17.39	11.99	7.50	11.76	5.64
CPA	26.79	23.31	11.22	12.16	23.10
EUR	5.04	11.28	8.03	10.72	7.89
FSU		13.46	4.40		
LAM	14.65	19.07	10.11	15.08	9.10
MEA	10.61	11.74	4.17	8.74	1.36
NAM	23.10	10.60	8.54	8.78	8.99
PAO	-5.58	13.05	11.91	13.23	11.19
PAS	14.19	19.39	11.66	14.85	17.64
SAS	4.82	16.33	9.69	14.82	8.37

**Table 8:** Significant centennial trends (values only shown for p < 0.05) for animal-based demand shares (kcal kcal^−1^ capita^−1^ day^−1^ 0.01 a^−1^) between 1988 and 2007 for FAO data and between 1990 and 2010 for all scenarios estimated using the Mann-Kendall test.

Region	FAO	A1	A2	B1	B2
AFR		0.12	0.06	0.14	0.02
CPA	0.58	0.40	0.16	0.30	0.63
EUR	-0.11	0.06	-0.02	-0.08	-0.11
FSU	-0.33	0.24			
LAM	0.18	0.26	0.07	0.27	0.13
MEA	0.09	0.19	0.09	0.19	0.07
NAM	-0.07	0.04		-0.17	-0.18
PAO	-0.03	0.07	0.06	-0.10	-0.05
PAS	0.10	0.24	0.12	0.23	0.27
SAS	0.13	0.18	0.09	0.22	0.08

**Fig 5:**
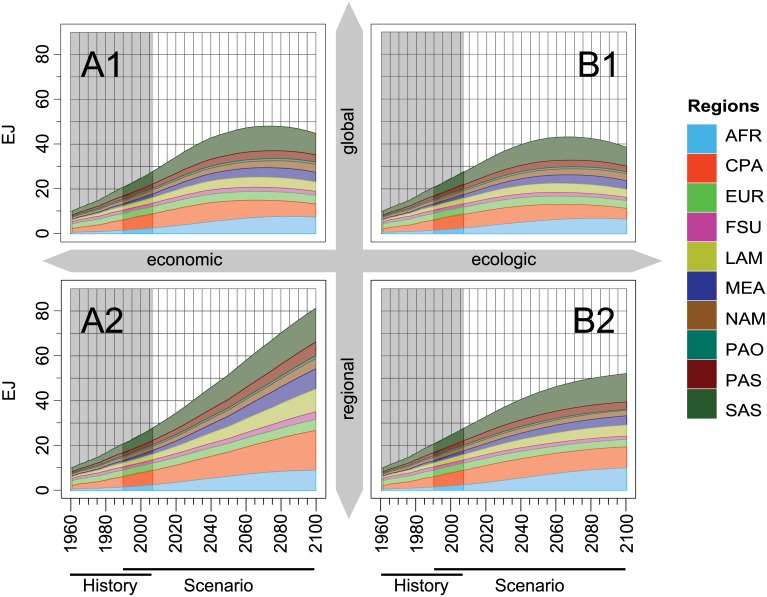
Total food energy demand projections per region over time for 4 SRES scenarios (10^18^ Joule a^−1^). Figures consist of historical data by FAO (1961–2007, “History”), respective projections (1990–2100, “Scenario”).

Regionally aggregated values (population-weighted averages) show a strong increase in total calorie demand up to 2050 in all scenarios (Fig 5). In the second half of the century, the scenarios diverge with either a continuous increase (A2), a stagnation (B2) or a slight decrease (A1, B1). Population growth (Figure A in S2 Fig), especially in developing countries, is the major driver for food demand throughout all scenarios. World population grows by 65% from 1990 to 2050 in the A1 and B1 scenarios, by 78% in the B2 scenario and by 114% in the A2 scenario. Compared to population growth, the increase in per-capita demand has a lower, yet substantial influence on total demand. It grows by 18% (A2), 21% (B2), 26% (B1) and 36% (A1) from 1990 to 2050. Here, the regions with the highest growth rates in per-capita demand are Sub-Saharan Africa, South, Central as well as South-East Asia.

The high per-capita demand values by far exceed plausible intake averages over the population of a country. As the Food Balance Sheets food availability also includes household waste, a substantial share of the food demand has to be interpreted as waste. Already today, high shares of the food demand can be attributed to waste in developed countries [1].

**Fig 6:**
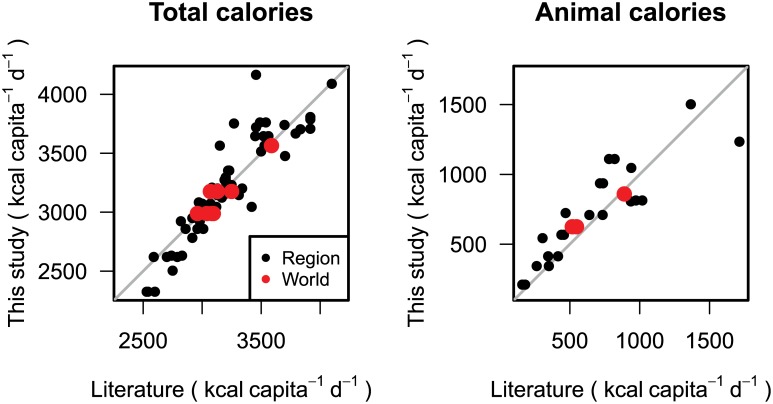
Comparison of estimates of total (left) and animal-based (right) calorie demand between values from the literature [17, 19–21] (x-axis) and own calculations (y-axis). Values are given for 2030 and 2050 as regional means (black dots) which are derived as population weighted averages of countries in the same regions as given in the literature and global means (red dots). Compare Tables (9, 10 and 11) and discussion in section on “Comparison of calorie demand projections to other studies”.

In order to compare our results with other studies [17, 19–21], we aggregated the country-based values of the respective scenario to the same regions as given in the literature. These regional population-weighted means as well as global averages compare well (Fig 6, left and Tables 9, 10 and 11).

**Table 9:** Comparison of projections in kcal capita^−1^ d^−1^. Transforming the projections of Alexandratos et al. [18] for animal products into calorie values has been done by Valin et al. [19]. We aggregated our national values in order to match the FAO regions below. In comparison with values from FAOSTAT [1], we presume that (Table 2, [19]) refers to total animal-based calories. The B2-Regional scenario of Valin et al. [19] takes population and income projections of the B2-scenario of Nakicenovic et al. [8] and assumes that food demand evolves according to strong regional specifications. DC = Developing Countries, IC = Industrial Countries (including Australia, Japan and New Zewland), TC = Transition Countries. The following subregions belong to DC: SSA = Sub-Sahara Africa (excluding South Africa), MEA = Middle East and North Africa (including Turkey and Afghanistan), LAM = Latin America, SAS = South Asia, EAS = East Asia.

	Alexandratos et al. 2006 [18]		Valin et al. 2010 [19]		Alexandratos and Bruinsma 2012 [17]	This study	
Scenario	Default		B2-Regional		Default	B2	
	Total	Animal	Total	Animal	Total	Total	Animal
**Year 2030**							
**World**	**3040**	**548**	**3095**	**518**	**2960**	**2990**	**626**
**DC**	**2960**	**458**	**3010**	**440**	**2860**	**2859**	**568**
SSA	2600	180	2541	160	2530	2326	211
MEA	3130	416	3311	345	3130	3146	414
LAM	3120	735	3420	640	3090	3046	710
SAS	2790	350	2701	263	2590	2621	344
EAS	3190	738	3339	716	3130	3202	937
**IC**	**3520**	**1019**	**3562**	**973**	**3450**	**3646**	**814**
**TC**	**3150**	**821**	**3529**	**781**		**3565**	**1111**
**Year 2050**							
**World**	**3130**				**3070**	**3177**	**702**
**DC**	**3070**				**3000**	**3073**	**683**
SSA	2830				2740	2632	363
MEA	3190				3200	3274	552
LAM	3200				3200	3296	805
SAS	2980				2820	2924	563
EAS	3230				3220	3354	994
**IC**	**3540**				**3490**	**3762**	**697**
**TC**	**3270**					**3753**	**1077**

**Table 10:** Comparison of projections in kcal capita^−1^ d^−1^. Values extracted from Kruse [21]. We aggregated our national values in order to be comparable. ME = Middle East (including Turkey), LAM = Latin America, Asia (including Afghanistan), FSU = Former Soviet Union, EUR = Europe, NAM = North America.

	Kruse 2010 [21]	This study
Scenario	Default	B2
	Total	Total
**Year 2030**		
**World**	**3083**	**2990**
Africa	2750	2505
ME	3167	3124
LAM	2958	2955
Asia	2917	2951
FSU	3500	3516
EUR	3833	3704
NAM	3792	3668
**Year 2050**		
**World**	**3250**	**3177**
Africa	2917	2783
ME	3250	3231
LAM	3083	3209
Asia	3208	3160
FSU	3917	3708
EUR	3917	3808
NAM	3917	3788

**Table 11:** Comparison of projections in kcal capita^−1^ d^−1^. We aggregated our national values in order to match the regional values. The AGO scenario (Agrimonde Global Orchestration) is based on the Global Orchestration scenario of the Millenium Assessment report. SSA = Sub-Sahara Africa (including South Africa), MEA = Middle East and North Africa (including Turkey and Afghanistan), LAM = Latin America, Asia (including Afghanistan), FSU = Former Soviet Union, OECD (including Turkey).

	Dorin et al. 2011 [20]		This study	
Scenario	AGO		A1	
	Total	Animal	Total	Animal
**Year 2050**				
**World**	**3588**	**890**	**3566**	**861**
SSA	2972	305	3084	544
MEA	3457	470	3721	724
LAM	3698	940	3741	1047
Asia	3703	937	3477	807
FSU	3457	1366	4166	1503
OECD	4099	1714	4090	1235

### Projections for the animal-based calorie demand

Animal-based calorie demand (S4, S5, S6 and S7 Figs) increases for all countries and scenarios only until the middle of the 21^*st*^ century. Here, some countries with a traditionally high share of meat in the diet (e.g. Mongolia or Hungary) reach a per-capita animal-based calorie demand of more than 2000 kcal capita^−1^ d^−1^. Projections for the end of the century show similarities between A1 and A2 as well as between B1 and B2 caused by the application of the same function but are not identical because of differing income projections. The highest overall deviations between scenarios are projected for countries with traditionally high meat consumption (like Russia, eastern Europe or Northern America) with dramatic decreases of demand values for animal-based products. Also for countries with very low historical values (like most countries in Africa), the differences for 2100 are high both due to the income projections and the applied regression functions.

**Fig 7:**
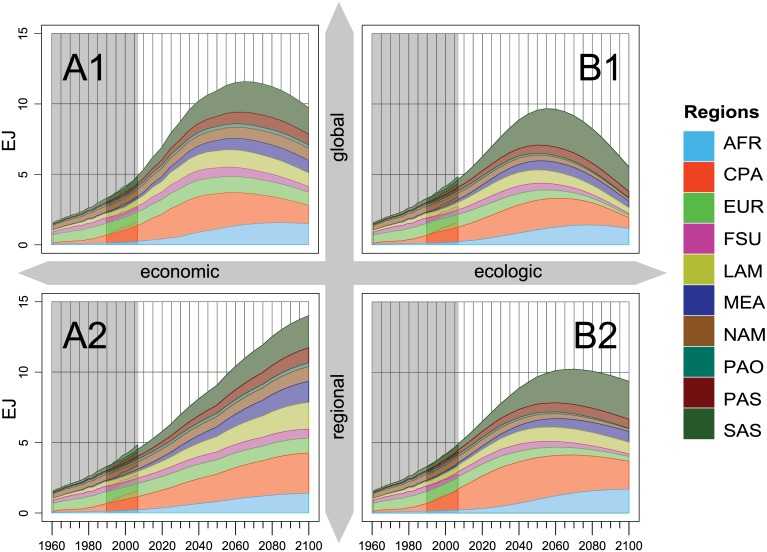
Same as Fig 5 for animal-based food energy demand projections per region over time for 4 SRES scenarios (10^18^ Joule a^−1^).

The regionally aggregated demand for animal-based products increases in all four scenarios (Fig 7). With global growth rates of 176% (A2), 191% (B1), 194% (B2) and 233% (A1), it outpaces the average increase in total food consumption. The increase is strongest in Sub-Saharan Africa where total animal-based demand in 2050 reaches 7 to 9 times the value of 1990. In South Asia, demand also increases by a factor of 5 to 9. In contrast, animal-based demand stagnates in the OECD regions in the scenarios A1 and A2 or even declines in the environmentally oriented scenarios B1 and B2.

For animal-based demand in the A1 and B1 scenarios, the increase in per-capita demand by 106% (A1) and 80% (B1) becomes the dominant driver, compared to the increase in population. In the regionalized A2 and B2 scenarios, population growth still remains the dominant driver, but also per-capita demand grows by 31% (A2) and 68% (B2). Throughout all four animal-based demand projections, the increase in the share of animal-based calories is more decisive than the increase in total calorie demand per capita.

In the second half of the century, most regions already have a decreasing share of animal-based calories. Only in the least developed regions like Sub-Saharan Africa, South and South-East Asia, the animal-based share in some scenarios still slowly increases. At the same time, global demand for animal-based calories decreases in all but the A2 scenario (Fig 7). In the B scenarios this can be explained by a declining share and stagnating per-capita calorie consumption. In the globalized scenarios A1 and B1, a decreasing global population also contributes to the decline in the demand for animal-based products.

Comparing our results to those studies with projections for animal-based calories [19, 20], the regional population-weighted means as well as global averages are also in good agreement (Fig 6, right and Tables 9 and 11).

## Validation

The resulting demand values are examined from three different angles: their sensitivity towards the data used for building the regression models, their trends in the common period of data and projections and their comparison with values reported in the literature.

### Sensitivity of demand regression models

Each of the regression models was tested with regard to the sensitivity of the estimated parameters on the data used for the fit (as described in the Methods section). By randomly sampling a subset of the data for 1000 times and fitting the regression with this subset, the robustness of the models can be evaluated by two criteria. On the one hand, the parameters derived from the subset can be compared to those from the entire dataset, and on the other hand, the resulting estimates for total calorie demand and the animal-based calorie share can be compared to the residual data subset that were not used for the fit.

For the first criterion, we divided the parameters determined from the respective subset of the data set through the parameters determined with the entire data set as the relative deviation from one can serve as criterion for the evaluation of model robustness. Our results show that for all parameters the relation between the parameters derived from the subset and those derived from the entire data set is close to one (Figures A and C in S11 and S12 Figs). Slight deviations can be seen for parameter *ω*_2_ for model *g*_*B*_ (Figure C S11 Fig). The second criterion is evaluated by using the residual third part of the dataset not used for the fit and calculating the coefficient of determination of the linear regression between models and data. The distribution of the respective coefficient of determination (Figures B and D in S11 and S12 Figs) is very close to the one determined from the entire data set. So, both criteria support the credibility of the chosen regression models.

### Comparison of trends in overlapping period

The SRES projections start in 1990 and have an overlapping period of approximately 20 years with historical data. Within this period, population and income projections already diverge substantially from historical records (S1 Text). Nevertheless, we decided to keep the base year of 1990 in order to make our results consistent with other SRES-based studies. In order to evaluate the temporal trends of the historical data in comparison to those from the scenarios, we calculate trends from datasets from the FAO (1988 to 2007) and from regression results (1990 to 2010) (see description of Mann-Kendall test in Methods section).

For most of the regions, the reported data show significant trends as calculated by the Mann-Kendall test. For the total calorie demand (Table 7), regions with highest trends are best met by globally oriented scenarios (A1 or B1), whereas medium increases are mostly met by the regional scenarios (A2 or B2). Trends for the animal-based share have much lower values (Table 8) and are in most cases well represented by regional scenarios. Here, the higher positive trends follow mostly an economic storyline (A2) and the lower and negative trends are better met by an ecologic scenario (B2).

### Comparison of calorie demand projections to other studies

In the following, we compare the projections of per-capita calorie demand to published estimates. Since the respective studies use world regions which partly differ from our geographical units, we aggregate our national values in order to make them comparable. For the comparison to Alexandratos [47], Valin et al. [19], Alexandratos and Bruinsma [17], and Kruse [21], we choose calorie projections of the B2 scenario (Table 9 and 10). For Valin et al. [19] this is a natural choice, since their assumptions on income and population are also based on the B2 scenario of Nakicenovic et al. [8]. The B2 scenario is also the most appropriate “middle of the road” for the comparison with Alexandratos [47], Alexandratos and Bruinsma [17] and Kruse [21], which have only one projection. To compare our calorie demand values to Dorin et al. [20] (Table 11), we choose the A1 scenario, which fits best to the Agrimonde Global Orchestration Scenario based on the Millenium Assessment report.

For global average per-capita calorie demand in the year 2000, other studies give values of 2789 kcal capita^−1^ d^−1^ [47], 2731 kcal capita^−1^ d^−1^ [19] and 2708 kcal capita^−1^ d^−1^ [21]. As these studies have the same base year, namely 2000, the difference between the studies is probably caused by different download dates from FAO food balance sheets on which their values are based. The base year of our study is 1990, values for 2000 are therefore projections, which at 2736 kcal capita^−1^ d^−1^ meet the global values of the other studies quite well.

Our global estimate for per-capita calorie demand in 2030 is within a range of 100 kcal capita^−1^ d^−1^ of Alexandratos [47], Valin et al. [19] and Kruse [21] for the B2 scenario. Valin et al. [19] also report global estimates for other scenarios which are comparable to our values. While they project 2995, 3095 and 3135 kcal capita^−1^ d^−1^ for the scenarios A1, B2 and B1, we predict 2949, 2990 and 3062 kcal capita^−1^ d^−1^, respectively. In 2050, our scenario B2 lies at 3177 kcal capita^−1^ d^−1^ between the results of Kruse [21] at 3259 kcal capita^−1^ d^−1^ and Alexandratos [47] at 3130 kcal capita^−1^ d^−1^. Our A1 scenario estimate for 2050 is only 20 kcal capita^−1^ d^−1^ lower than Dorin et al. [20]. On a regional level, the results of our B2 scenario for per-capita calorie demand are in between the estimates of Alexandratos [47], Kruse [21] and Valin et al. [19] for most regions. Only for Sub-Saharan Africa our estimates are lower, and for the transition countries of the Former Soviet Union we have slightly higher estimates.

Turning to animal-based demand, our global B2 estimate for 2030 is about 100 kcal capita^−1^ d^−1^ higher than in Valin et al. [19] and Alexandratos [47]. This is due to the temporal increase in animal-based demand in developing countries. The livestock share is 20.9% of the total demand in our study, while it is only 15.5% and 14.6% for Alexandratos [47] and Valin et al. [19]. On a regional level, we find that Valin et al. [19] and Alexandratos [47] project an increase in animal-based calories up to 2030 for the OECD regions, while our study projects a decrease. When comparing our results for animal-based calorie demand in 2050 to Dorin et al. [20], we have considerably lower estimates for the OECD countries. Therewith, our projection is able to reproduce the historical trend in North America, Europe and Pacific OECD, where the share of animal-based calories fell since the 1960s, 1980s and 1990s, respectively, and where the absolute amount of livestock calories stagnates or decreases. This pattern is most predominant in our sustainable scenarios B1 and B2, while e.g. in our global and materialistic A1 scenario animal-based calories in OECD regions increase to 1181 kcal capita^−1^ d^−1^ in 2030. Only after reaching a peak value of 1235 kcal capita^−1^ d^−1^ in 2050 they do start to decline.

For countries in transition we project quite a steep increase in animal-based calorie demand until the year 2030 compared to other studies. Traditionally, countries in Central and Eastern Europe and Former Soviet Union had very high livestock demand in relation to their income [48]. Due to our calibration to the base year 1990, this regional peculiarity persists in our approach into the near future. Furthermore, for the B2 scenario we assume that the animal-based calorie share is highest for medium incomes (function *h*_*B*_) which applies to Former Soviet Union countries.

### Child mortality and children underweight

**Fig 8:**
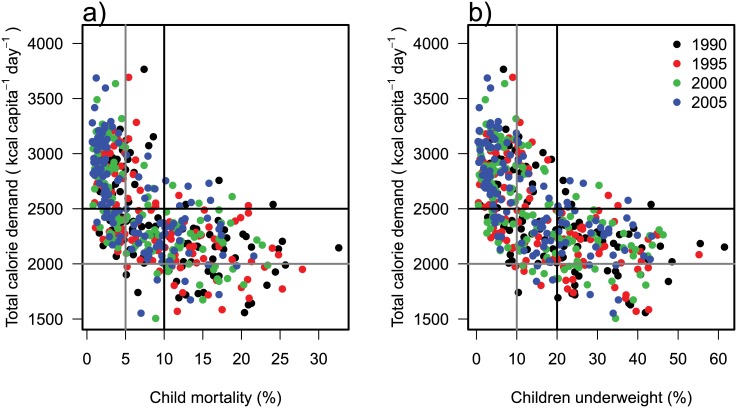
Percentages of child mortality (a) and children underweight (b) against food demand for 4 years (denoted by colors). Data are taken from Von Grebmer et al. [49] and FAOSTAT [1]. Lines indicate the thresholds for calorie demand of 2000 and 2500 kcal capita^−1^ d^−1^, child mortality percentages of 5 and 10% and children underweight percentages of 10 and 20%.

In order to relate food demand to food security, we refer to the independently estimated children mortality and children underweight indicators for children below 5 years. In countries reporting more than 2500 kcal capita^−1^ d^−1^, child mortality and children underweight is comparatively low. Only less than 3% of these countries have child mortalities of more than 10% (Fig 8a) and children underweight of more than 20% (Fig 8b). On the other hand, countries with less than 2000 kcal capita^−1^ d^−1^ report at least a child mortality of 5% and children underweight of 10%. Although calorie availability seems to be a good indicator for the lower end of both aspects of food security, the highest values for child mortality and children underweight are not reported for countries with the lowest calorie demand values. More than every fourth child under five dies in African countries (Mali, Niger, Sierra Leone or Rwanda), where demand values between 1780 and 2200 kcal capita^−1^ d^−1^ are calculated. And nearly every second child (> 45%) under five is underweight in Asian countries (Bangladesh, India, Nepal or Cambodia) although demand values are above 2000 kcal capita^−1^ d^−1^ with one exception (between 1850 and 2300 kcal capita^−1^ d^−1^).

## Discussion

### Interpretation of functional relations

Driving processes behind food demand dynamics are most likely different for the short-term and for the long-term. Major drivers of short-term dynamics include harvest failure, food storage capacities, expectation failures, policy interventions like trade regulations or biofuel targets, or business cycles and speculation. In contrast, major drivers of long-term dynamics include population, income, urbanization, demographic composition, market access, lifestyles, or the extent of physical labor. Long-term models should therefore consider other drivers and dynamics than short-term models. We decided to reduce the complexity to three major indicators that are representatives for these drivers, namely population, income and time.

Both sets of functions for per-capita consumption (*g*_*A*_ and *g*_*B*_) and the share of animal-based products (*h*_*A*_ and *h*_*B*_) give satisfactory results for the reproduction of historical data but assumptions on the form of the temporal development have a decisive effect on the projections. Especially the functional types for the share of animal-based products strongly diverge for high-income countries. As only 10 countries reach an average income above 40,000 US$ before 2005, the few observations do not allow to favor one of these functional relations.

Our statistical analysis cannot reveal the underlying causal relationship between income, time and food demand, which is open for debate. The strong increase of both total food demand and the share of animal-based products for low and medium income countries may be attributed amongst others to a higher purchasing power of consumers or a stronger economic development of food producers leading to falling prices for staple goods [5, 50]. The positive non income-related time trend of total food demand could stem from a higher social alienation from the food production process due to urbanization, more single member households and increased openness to global markets [5, 51]. Finally, the negative time-trend for the share of animal calories and the eventual negative connection to income could be attributed to a higher health consciousness among better-educated people [36] or to alternative lifestyles like vegetarianism becoming a status symbol.

At the same time, our empirical findings are not in line with the underlying assumptions of simple economic demand models that are based on a positive elasticity constant over time and income. The significant time trends in all our representations indicate that either elasticities should be adjusted over time, or that demand models should include other drivers than income. Additionally, our U-shaped regression even opens up the possibility to question a positive elasticity as such.

Our demand projections are influenced by our assumption, that national peculiarities, represented by the divergence of current demand from the regression results, disappear until the end of the 21^*st*^ century and that all values converge to the regression results (Eq 13). A contrasting hypothesis could be that these peculiarities are kept over time, such that the projections for 2100 deviate by the same percentage from the regression results as in the inital year (here 1990). Changing our assumption (Eq 13) accordingly leads globally to an increase of the total calorie demand of about 3.3% (2050) and 6% (2100). The alteration of the assumption would have larger consequences on the heterogeneity across countries. Whereas under the convergence assumption, the standard deviation across countries is about 704 kcal capita^−1^ d^−1^; this value increases to 833 kcal capita^−1^ d^−1^ when the peculiarities in national diets are maintained. Total demand is much higher than in the convergence estimates for the former Soviet Union members like Uzbekistan, Kosovo, Bosnia and Herzegovina, Serbia, Montenegro, or Ukraine, while it is lower for African countries like Angola, Chad, Eritrea, or Ethiopia. Nevertheless, the analysis of the FAO statistics of the past 50 years already reveals a convergence of food baskets [52] so that the assumption of convergent diets is most likely.

### Interpretation of projection results

For long-term projections, data quality is still low and the understanding of socio-economic dynamics is weak. Future analysis has therefore to rely on the evaluation of multiple scenarios instead of best-guess forecasts. The benefit of such scenario analysis is to consistently deliberate the implications of a set of assumptions. We provide a tool that can be used for such scenario analysis. Even though the set of adjustable assumptions is limited (adjustable population and per-capita income, two sets of functional relations for per capita caloric demand and for the share of livestock-based calories, two types of calibration, exogenous values for long-term targets), this provides already considerable freedom to create own scenarios that can be adapted to a storyline consistently.

Exemplarily, we applied our method to the SRES storylines, which are characterized by the contrasting concepts of economic (A) versus ecological values (B) and increasing globalization (1) versus regionalization (2). Their combinations result in different developments regarding population increases (Figure A in S2 Fig) as well as income projections (Figure B in S2 Fig).

The resulting regional per-capita demand values remain in reasonable ranges given the strong income growth in the scenarios. The highest value for total calorie demand of 4700 kcal capita^−1^ d^−1^ for Northern America (S8 Fig) has to be seen in the context of the materialistic storyline of scenario A1 and a per-capita income which quadruples compared to current levels. For the sustainable scenarios B1 and B2, the projected per-capita demand exceeds only slightly the observed range of the past despite more than doubling per-capita income. Animal-based calorie demand on the regional level (S9 Fig) remains completely in the observed range. The increase of vegetal demand beyond observed ranges (S10 Fig) in scenarios B1 and B2 stems from the reduction of animal-based calorie demand due to the assumption of a more healthy diet in higher income ranges.

Some of our results seem surprising given the SRES storylines, but are consistent at a closer look.

First, one would not expect an increase of per-capita demand in high-income countries, as metabolic requirements are constant or even decline due to less physical labour in high-income groups [4]. Nevertheless, demand in these regions still increases, which has to be attributed to an increase in food waste at the household level.

Second, it seems plausible that food demand in the environmentally oriented scenarios (B1 and B2) (S6 and S7 Figs) is lower than in the materialistic oriented scenarios (A1 and A2) (S4 and S5 Figs) as people according to the latter storylines are likely to waste more food. A closer look reveals that a highly populated A2 world goes along with lower per-capita incomes in many developing regions. This leads to lower total per-capita demand values and, hence, people simply cannot afford to waste food.

Third, the animal-based share in developing countries increases faster in the environmental than in the materialistic scenarios. This occurs only for medium income countries with an income range that increases from 1,000–26,000 US$ in 1990 to 3,600–39,000 US$ in 2100. In this income range, the projected livestock share is smaller for function *h*_*A*_ (applied to A1 and A2) than for *h*_*B*_ (applied to B1 and B2). For the same income and year, results from function *h*_*A*_ are up to 20% lower than values from *h*_*B*_. Especially for the regionalized scenario B2, income in many countries stays below 30,000 US$ so that their livestock share is higher than in scenarios A1 and A2. This peculiarity comes from the requirement that both models have to be consistent with past data. If the share of animal-based calories is assumed to fall with high incomes, the medium incomes need to have an even higher share in order to fit observations (compare blue curves in Fig 4 with red curves). We did not find a functional type fitting the same observation data that produces lower results for every possible combination of drivers.

Although using a single global approach for the estimation of food demand on the country level, trends of the projections in the overlapping period of data and projections compare well to those from observed data (Tables 7 and 8). Moreover, our estimates are well within the range of published projections by other authors (see results section). Finally, we showed with an analysis of regression models with random subsamples of two thirds of the observations that the other third can be predicted with a similar coefficient of determination as the model from the entire dataset (S11 and S12 Figs).

### Food security prospects

The share of population which is undernourished declines with increasing per-capita demand at the country-level [49, 53]. According to FAO [53], countries with more than 2500 kcal capita^−1^ d^−1^ do not observe undernourishment for more than 20% of the population. However, estimating the prevalence of undernourishment based on distribution corrected food availability as done by FAO [53] was criticized as being unreliable because it is a top-down approach that has to rely on poor statistical data [54]. Instead, Svedberg [54] proposes to use anthropometric bottom-up measurements like the prevalence of stunting and underweight. Unfortunately, global data on food security is still deficient and incomplete [55]. One of the few available global datasets that includes anthropometric measurements is the Global Hunger Index (GHI) [49] which is composed to equal shares of the prevalence of child underweight, child mortality and FAO’s [53] top-down estimates. The GHI shows surprisingly strong correlation (Pearsons correlation coefficient of -0.84) with average per-capita demand per country [1] but it has to be considered that the data sources are not independent as undernourishment is estimated partly based on FAOSTAT [1].

According to our projections, the range of values between 2000 and 2500 kcal capita^−1^ d^−1^ will persist in some countries also in the coming decades (S4, S5, S6 and S7 Figs). While there is no country in 2050 that has a per-capita demand below 2000 kcal capita^−1^ d^−1^, many people still live in countries with an average demand of 2000–2500 kcal capita^−1^ d^−1^, ranging from 1.9 billion people in the A2 scenario to 0.3 billion people in the A1 scenario.

The major geographical areas of concern are clustered in the tropics. Most severely affected are the world regions Sub-Saharan Africa (< 2400 kcal capita^−1^ d^−1^ and 8% animal-based calories in the year 2005) and South Asia (< 2300 kcal capita^−1^ d^−1^ and 9% animal-based calories in 1990). According to our scenarios, per-capita demand will grow slowly in both regions. Comparing the demand in these least developed regions to the demand of a transition country like China in 1990 (≈ 2600 kcal capita^−1^ d^−1^, 12% animal-based calories), Sub-Saharan Africa will reach a similar level only in 2030 (A1) or 2045 (B2). Also South Asia will reach this level only between 2020 (A1) and 2030 (A2). This suggests a time lag of 30 to 55 years to the development of China.

While food availability can be expected to increase, also obesity and related health problems are currently strongly rising. Already today, obesity is responsible for a similar extent of pre-mature deaths as hunger [56]. It is of large interest to include the dynamics that determine overnourishment into future assessments.

### Conclusions

This paper provides a simple method to create customized food-demand scenarios. It requires only information on gross domestic product and population which are the key indicators of many future scenarios. Despite this slim approach, the results of our projections are in good accordance with other studies.

Many investigations use scenarios that are adapted ex post to meet a certain storyline, e.g. by reducing the demand of livestock products by a certain share [10, 11, 57]. Our method is exceptional by adapting ex ante the core assumptions of the functional relations instead of changing the results ex post. In so doing, projections remain consistent with historical data. The results of our ex-ante customization sometimes seem unintuitive at the first glance, but at a closer look they are logical and more consistent with the overall storyline and history than an ex-post approach.

Our method has been applied exemplarily for constructing a set of food demand scenarios consistent with the SRES storylines, but could also be easily used for upcoming scenarios like the Shared Socio-economic Pathways (SSP, [58]). Applying our method or using our results may be of interest for different scientific communities. For investigations of food security aspects, our long-term scenarios suggest that food availability will rise above critical thresholds for wide parts of the currently developing countries. However, food availability in some countries, in particular in Sub-Saharan Africa, is likely to remain at critical levels in the coming decades such that long-term engagement is required. For health studies, the dynamics of excessive consumption of animal-based calories may be interesting, as it can be clearly linked to a number of heart and vascular health problems and colon cancer [59–63]. Finally, our long-term food demand scenarios can be valuable for the environmental science community. As our results suggest a doubling of food demand in all scenarios and an over-proportional increase of animal-based calorie demand, the pressure on environmental systems can be expected to increase strongly.

The model used in our study is provided as a package in the programming language R. We hope that other researchers will make use of and extend this open source project. For example, future research could try to split food demand projections into intake and waste. This could be done by estimating food energy requirements of a population based on body mass index, age structure, and physical activity, correcting for over- and under-consumption with estimates on stunting and obesity. Also, the model could be extended to include lifestyle-related drivers of food demand like urbanization or education for which historical panel datasets exist.

## Supporting Information

S1 Text Provision of programs and visualization tools on GitHub. (PDF)

S2 Text Discussion on data sources. (PDF)

S1 Fig World regions. World regions with similar socio-economic conditions according to [44]: AFR = Sub-Sahara Africa, CPA = Centrally Planned Asia (incl. China), EUR = Europe (incl. Turkey), FSU = Former Soviet Union, LAM = Latin America, MEA = Middle East, NAM = North America, PAO = Pacific OECD (Australia, Japan and New Zealand), PAS = Pacific Asia, SAS = South Asia (incl. India).
(EPS)

S2 Fig Regional population and income projections for SRES scenarios. **Population (Figure A) and per-capita income projections (Figure B)** of the four SRES scenarios aggregated to 10 world regions (S1 Fig) in 10^9^ capita and in 10^3^ US$_2005_ capita^−1^.
(EPS)

S3 Fig Regression parameter values. Regression parameter values for total calories with functions *g*_*A*_, *g*_*B*_ and *h*_*B*_. Parameter α* (left) and *β* (right) for functions *g*_*A*_ and *g*_*B*_ (**Figure A**). Yearly values of the linear mixed effect model (y1: black dots) and the linear regression (Table 2) (r1: black line) are given as well as yearly fitted values for the non-linear fit (y2: grey diamonds) and the Michaelis-Menten-type functions (Table 4) (r2: grey line). Regression parameter values for total calories with function *h*_*B*_ for parameter *ρ* (left) and *λ* (right) (**Figure B**). Yearly fitted values for the non-linear function (dots) and the linear regression (Table 6) (line) are given.
(EPS)

S4 Fig Country-specific projections of SRES scenario A1. **Maps of projections** for total (left) and animal-based (right) calories per capita for the years 2005 (middle), 2050 (middle) and 2100 (bottom).
(EPS)

S5 Fig Country-specific projections of SRES scenario A2. **Maps of projections** for total (left) and animal-based (right) calories per capita for the years 2005 (middle), 2050 (middle) and 2100 (bottom).
(EPS)

S6 Fig Country-specific projections of SRES scenario B1. **Maps of projections** for total (left) and animal-based (right) calories per capita for the years 2005 (middle), 2050 (middle) and 2100 (bottom).
(EPS)

S7 Fig Country-specific projections of SRES scenario B2. **Maps of projections** for total (left) and animal-based (right) calories per capita for the years 2005 (middle), 2050 (middle) and 2100 (bottom).
(EPS)

S8 Fig Regional projections of total calorie demand scenarios. **Total calorie demand projections** for 4 SRES scenarios as population-weighted means for each world region. Shaded areas indicate observed value ranges from historical FAO data on the country (light shading) and on the regional scale (dark shading).
(EPS)

S9 Fig Regional projections of animal-based calorie demand scenarios. **Animal-based calorie demand projections** for 4 SRES scenarios as population-weighted means for each world region. Shaded areas indicate observed value ranges from historical FAO data on the country (light shading) and on the regional scale (dark shading).
(EPS)

S10 Fig Regional projections of vegetal-based calorie demand scenarios. **Vegetal-based calorie demand projections** for 4 SRES scenarios as population-weighted means for each world region. Shaded areas indicate observed value ranges from historical FAO data on the country (light shading) and on the regional scale (dark shading).
(EPS)

S11 Fig Sensitivity of regression models g_*A*_ and g_*B*_. **Sensitivity analysis of regression model parameters** with respect to the chosen data subsample. Boxplots of ratios of parameters for function g_*A*_ (Figure A) and function g_*B*_ (Figure C) from 1000 subsamples of the dataset to the parameters derived from the entire dataset. Orange line denotes unity and indicates a perfect fit. Distribution of coefficient of determination of linear regression based on non-chosen part of the dataset between data and model result for function g_*A*_ (Figure B) and g_*B*_ (Figure D). Orange lines depict the coefficient of determination of the regression model based on the entire dataset.
(EPS)

S12 Fig Sensitivity of regression models h_*A*_ and h_*B*_. **Sensitivity analysis of regression model parameters** with respect to the chosen data subsample. Boxplots of ratios of parameters for function h_*A*_ (Figure A) and function h_*B*_ (Figure C) from 1000 subsamples of the dataset to the parameters derived from the entire dataset. Orange line denotes unity and indicates a perfect fit. Distribution of coefficient of determination of linear regression based on non-chosen part of the dataset between data and model result for function h_*A*_ (Figure B) and h_*B*_ (Figure D). Orange lines depict the coefficient of determination of the regression model based on the entire dataset.
(EPS)

S1 Dataset SRES scenario results of food calorie demand. **Datasets of the historical period and SRES scenario projections** are included in a Excel file. The data are available on country and regional resolution for total and animal-based food calorie demand as well as for income and population data.
(ZIP)
