# Supplementary material for: Global Food Demand Scenarios for the 21st Century
Source: PLoS One. 2015 Nov 4;10(11):e0139201. doi: 10.1371/journal.pone.0139201 (PMC4633131; doi:10.1371/journal.pone.0139201)
Supplement: S1 Text — (PDF) [file pone.0139201.s001.pdf]

Provision of programs and visualization tools

We provide a visualization tool and the open source code for our projections.

The open source R package "CalorieDemand" contains functions and input data of our approach which are described in help files and a manual. These can be used to create own scenario estimates for total and animal-based calorie demand. The package is written in the programming language R [1]. The website Demand Scenarios (http://www.pik-potsdam.de/demand-scenarios) provides information on the ongoing model development of the open source project and the web-based visualization tool. The open source model as used in this publication is archived at Food Demand Model (https://github.com/bodirsky/Food_demand_model/releases/tag/1.0/, doi:10.5281/zenodo.31008) and the visualisation tool under Visualization Tool (https://github.com/bodirsky/Food_demand_visualisation_tool/releases/tag/1.0).

References

[1] R Development Core Team. R: A Language and Environment for Statistical Computing. ISBN 3-900051-07-0; 2012. Www.R-project.org.
