# Supplementary material for: Global Food Demand Scenarios for the 21st Century
Source: PLoS One. 2015 Nov 4;10(11):e0139201. doi: 10.1371/journal.pone.0139201 (PMC4633131; doi:10.1371/journal.pone.0139201)
Supplement: S2 Text — (PDF) [file pone.0139201.s002.pdf]

Discussion on data sources

Beside the uncertainty connected to our assumptions and methodology, the uncertainty underlying the data we use is also passed on to our estimates. While some random errors might cancel out in our method, systematic biases in a specific direction will persist. In the following we will discuss the quality of each data source.

FAOSTAT

FAOSTAT provides the most comprehensive statistical database on global agricultural production and use. According to [1], data is mostly collected based on interviews, surveys or secondary sources. Only about 10 percent of data in European countries is based on direct physical measurements. In developing countries, data availability is even worse and data gaps have often been filled using extrapolation techniques. [1] estimates, that errors in country data might be as much as 20-25%, and global phytomass may diverge by 5-10 %. The subsequent use of produced crops and livestock products is even more uncertain than their production. Errors accumulate over the estimation of the shares used for feed, food, seed or processing, the postharvest and processing losses and nutritional contents of the final products. A large part of the underlying parameters are not obtained from national statistics but have to be estimated in the FAO headquarters in Rome [1].

Most errors probably do not represent a systematic bias in one direction. They may thus partly cancel out within the regressions. However, any systematic bias of our input data will remain in our regressions and thus in our scenario projections. One example for a systematic bias is that a number of food items hunted or gathered from wildlife (e.g. wild meat, insects or gathered plants, seeds, nuts and fruits) are not represented in these statistics [1]. As poor countries tend to have higher shares of products from wildlife origin, we systematically underestimate their food availability. Yet, as incomes increase in the future, the projections are more influenced by the data points with high income that tend to have a better data quality.

Worldbank Data

Also GDP estimates are subject to uncertainty. As GDP is usually estimated based on tax data, shadow markets and smuggled goods are not covered. Subsistence production, making up a major part of poor economies, is also not covered. In general, this leads to a systematic bias, underestimating the GDP of less developed countries. As some goods and services cannot be traded, the purchase power of a currency can also be different within the country to its purchase power abroad so that the use of GDP in purchase power parity (PPP) would be better comparable in the international context [2]. Nevertheless, we based our regressions on GDP in market exchange rates (MER) because of two reasons. Firstly, available Worldbank data for MER have a longer coverage (since 1960) than PPP values (since 1980). Secondly, the SRES GDP scenarios [3] are only available in MER.

Population estimates are also rather uncertain. Due to high administrative efforts population censuses are carried out only in larger time-intervals. 17 % of all countries in the UN 1998 Revision had no census data from after 1985 available [4]. Revised population estimates may therefore show substantial correction. For example, a census in Nigeria in 1991 came up with 35 million people less than expected [5].

SRES projections

The SRES population and income scenarios [6, 7] are calibrated to the year 1990 and therefore inherit the uncertainty of the base year estimates mentioned above. For the subsequent years 1990-2010, which lie now in the past, the SRES scenarios are already projections and therefore diverge substantially from recorded values [8]. The SRES projections overestimate global average GDP per capita in 2010 by 5% (B1) to 9% (A2) compared to [8]. Regional divergences are even higher. The per capita GDP in Centrally Planned Asia (CPA) was estimated to be 22% (A1), 58%(A2), 44%(B1) lower or 14% (B2) higher. For Middle East and North Africa (MEA), scenario values were too high by 22% to 85%. Errors are much lower in developed regions than in developing countries. Also the population scenarios diverge from recorded values [8]. In 2010, the projections for A1, B1 and B2 were approximately 1% higher, while A2 was almost 5% higher. Again, regional estimates for our 10 world regions are much higher with a spread of -3 to +11%. There is a clear tendency in all scenarios to overestimate population growth in developed countries and to underestimate the growth in developing countries.

The originally regional scenarios were disaggregated by [6, 7] to country level by applying the regional growth rates to country-specific starting values. This method creates unrealistic values for countries with relatively small population that diverge in their state of development from the rest of their region. Examples include Mauritius, The Bahamas, Singapore, or Macao, which reach extremely high values for per-capita income in 2100.

References

[1] Smil V. Feeding the World: A Challenge for the 21st century. The MIT Press; 2000. ISBN 9780262692717.

[2] Nordhaus W. Alternative measures of output in global economic-environmental models: Purchasing power parity or market exchange rates? Energy Economics. 2007;29(3):349 – 372.

[3] Nakicenovic N, Alcamo J, Davis G, de Vries B, Fenhann J, Gaffin S, et al. Special Report on Emissions Scenarios: a special report of Working Group III of the Intergovernmental Panel on Climate Change. New York, (US): Cambridge University Press; 2000. ISBN 0-521-80493-0.

[4] Zlotnik H. World Population Prospects: The 1998 Revision. Perugia, Italy: United Nations Population Devision; 1999.

[5] Keilman N. Data quality and accuracy in United Nations population projections 1950-95. Population Studies. 2001;55(2):149 – 164.

[6] CIESIN. Country-level Population and Downscaled Projections based on the B2 Scenario, 1990-2100; 2002. http://www.ciesin.columbia.edu/datasets/downscaled.

[7] CIESIN. Country-level GDP and Downscaled Projections based on the A1, A2, B1, and B2 Marker Scenarios, 1990-2100; 2002. http://www.ciesin.columbia.edu/datasets/downscaled.

[8] WORLDBANK. World Development Indicators; 2011. http://data.worldbank.org/data-catalog/world-development-indicators.
